# Discoverability of information on clinical trial data-sharing platforms

**DOI:** 10.5195/jmla.2021.992

**Published:** 2021-04-01

**Authors:** Paije Wilson, Vojtech Huser

**Affiliations:** 1 paije.wilson@wisc.edu, National Library of Medicine Associate Fellow, National Library of Medicine, Bethesda, MD (at time of study). Health Sciences Librarian, University of Wisconsin-Madison, Madison, WI; 2 vojtech.huser@nih.gov, Staff Scientist, National Institutes of Health, Bethesda, MD

**Keywords:** clinical trials, data-sharing platforms, data sharing

## Abstract

**Objective:**

This study was intended to (1) provide clinical trial data-sharing platform designers with insight into users' experiences when attempting to evaluate and access datasets, (2) spark conversations about improving the transparency and discoverability of clinical trial data, and (3) provide a partial view of the current information-sharing landscape for clinical trials.

**Methods:**

We evaluated preview information provided for 10 datasets in each of 7 clinical trial data-sharing platforms between February and April 2019. Specifically, we evaluated the platforms in terms of the extent to which we found (1) preview information about the dataset, (2) trial information on ClinicalTrials.gov and other external websites, and (3) evidence of the existence of trial protocols and data dictionaries.

**Results:**

All seven platforms provided data previews. Three platforms provided information on data file format (e.g., CSV, SAS file). Three allowed batch downloads of datasets (i.e., downloading multiple datasets with a single request), whereas four required separate requests for each dataset. All but one platform linked to ClinicalTrials.gov records, but only one platform had ClinicalTrails.gov records that linked back to the platform. Three platforms consistently linked to external websites and primary publications. Four platforms provided evidence of the presence of a protocol, and six platforms provided evidence of the presence of data dictionaries.

**Conclusions:**

More work is needed to improve the discoverability, transparency, and utility of information on clinical trial data-sharing platforms. Increasing the amount of dataset preview information available to users could considerably improve the discoverability and utility of clinical trial data.

## INTRODUCTION

Open science refers to the principle that all research methodologies, tools, documentation, data, and other material should be readily accessible to other researchers [1]. Full and complete sharing of research materials may be beneficial in a number of ways, such as by increasing confidence in research findings, facilitating collaborations among researchers, and advancing scientific innovation [2–5]. To realize these benefits, numerous funding agencies and journals have enacted policies to mandate or encourage the sharing of research data and, in many cases, its supporting documentation [5, 6].

Clinical trials involving humans are not excluded from pressure to share research materials. The International Committee of Medical Journal Editors (ICMJE) released a statement in 2017 requiring clinical trial researchers to submit a data-sharing statement and plan when submitting a manuscript to any ICMJE member journal [7]. Similarly, *The BMJ* and *PLOS Medicine* strictly require that clinical trial data be shared as a condition of a manuscript's publication [3]. The U.S. National Institutes of Health (NIH) also implemented policies that require the sharing of data from any NIH-funded clinical trial, and both the NIH and National Library of Medicine released strategic plans that emphasize FAIR (findable, accessible, interoperable, and reusable) data-sharing standards for all funded studies [8, 9, 10]. Proponents of clinical trial data sharing emphasize that the sharing of data and associated research materials can facilitate discovery, reduce trial redundancy, and promote transparency [6]. Some scholars also argue that it is essential to respect the altruism of trial participants by ensuring that their expectations of contributing to medical knowledge are maximally fulfilled, including through the sharing of research findings and associated data [7, 12, 13].

Despite the potential for greater discovery and transparency and the added ethical benefits of sharing clinical trial data, barriers to sharing and accessing clinical trial data persist. One such barrier is the wide variability in information provision across clinical trial data-sharing platforms, complicating the ability of users to choose a platform that suits their data-sharing or access needs [12, 14, 15]. This issue is exacerbated by the surplus of platforms in existence [5]. At a 2019 SAS webinar, “Exploring the Evolution of Data Transparency and Its Impact on Patient Outcomes,” representatives from clinical trial data-sharing platforms described similar challenges, emphasizing difficulties in enhancing the discoverability of their platforms and inspiring confidence in users wanting to access or submit data.

One way to address these issues is for clinical trial data-sharing platforms to provide sufficient summary information about particular datasets and their associated trials to allow users to evaluate their need for a dataset before expending effort to make a formal data request. In this study, we compared the availability of summary information for datasets on seven clinical trial data-sharing platforms: Clinical Study Data Request (CSDR), Vivli, Biolincc, dbGaP, Project Data Sphere (PDS), the National Institute on Drug Abuse (NIDA)'s Data Share, and the National Institute of Child and Health and Human Development (NICHD)'s Data and Specimen Hub (DASH). Specifically, we examined the extent to which platforms and individual dataset records provided preview information about data formats, access requirements, links to ClinicalTrials.gov and additional related resources, and the availability of protocols and data dictionaries. This study is intended to provide clinical trial data-sharing platform designers with insight into users' experiences when attempting to evaluate and access datasets, spark conversations about improving the transparency and discoverability of clinical trial data, and provide a partial view of the current information sharing landscape for clinical trials.

## METHODS

### Platform and dataset sample selection

Seven U.S. clinical trial data-sharing platforms were examined between February and April 2019. Only platforms for human clinical trial data that contained records for 50 or more trials were considered. We used a convenience sample of platforms based on consultation with clinical trial data-sharing experts and searching PubMed. Platforms were evaluated on the basis of their general characteristics and on a detailed review of a sample of 10 of the most recently submitted dataset records on each platform to mimic the experience of users who attempt to quickly evaluate a platform or dataset. If the first 10 datasets on a platform were from trials funded by the same sponsor, we chose the next most recent datasets from trials with different sponsors to ensure a degree of variability within the sample so that, whenever possible, at least two sponsors were represented within a platform. If a platform's dataset records could not be sorted by date, dataset records were selected at random.

In total, we evaluated 70 datasets. Some characteristics were evaluated at the dataset preview level, meaning that we only evaluated information present in the publicly viewable summary description of datasets and their associated trials and other resources (i.e., information available without performing a formal access request). Other, more platform-generalizable characteristics (e.g., whether users must pay to access datasets) were evaluated on the basis of whether they were easily discoverable on the platform's website without needing to consult several pages. A detailed description of our methods for evaluating characteristics within different categories is provided in the following sections.

### Dataset previews and access

Dataset previews and data access mechanisms are particularly important when deciding whether to request a dataset. Having access to the information needed to quickly determine the scope of the platform, the file formats of the datasets, and the effort required to obtain multiple datasets could help users determine the suitability of datasets for their own research purposes prior to investing the time to make a formal access request. Information on access requirements, such as request walls or paywalls, may also be useful in determining the amount of time and resources needed to access a dataset. Therefore, we evaluated the level of review (i.e., whether users can only view a summary description of a dataset or can fully download a dataset without the need for a formal access request), information on data formatting and file types, whether users need to make a single request or multiple requests to access multiple datasets, and the existence of request walls (i.e., data access is only possible after a formal request is submitted and approved) and paywalls for each platform.

### Links to ClinicalTrials.gov and other resources

Providing sufficient background information, such as links to clinical trial registries, primary publications, and sponsor websites, can better contextualize clinical trial datasets and enhance their discoverability. ClinicalTrials.gov is the largest clinical trial registry, containing records for 306,775 trials from 210 countries as of May 2019 [16]. Data registries such as ClinicalTrials.gov can increase the discoverability of datasets by data re-users, especially as ClinicalTrials.gov was created for the purpose of communicating timely and transparent results of clinical trials to the public [17, 18]. Therefore, we evaluated whether dataset previews had a link to their trial record in ClinicalTrials.gov (i.e., a platform-to-ClinicalTrials.gov link), whether dataset previews had a link from their trial record in ClinicalTrials.gov (i.e., a ClinicalTrials.gov-to-platform link), and whether the results of the trials associated with the datasets were posted on ClinicalTrials.gov. In addition to ClinicalTrials.gov, linking to other associated resources and literature can further enhance the contextualization of datasets [17, 19]. Therefore, links to other resources were evaluated by examining whether the dataset previews linked to non-ClinicalTrials.gov resources.

### Availability of protocols and data dictionaries

Protocols are vital resources for determining how data were collected, including information on the instruments and methods used and the overall data collection context, which can help researchers decide whether to reuse a dataset. Furthermore, making protocols available can deter selective reporting, promote greater understanding of data, and prevent unnecessary duplication of research [20, 21]. Data dictionaries provide highly granular details for each individual element within a dataset and are necessary for the proper reuse of data. Therefore, we examined whether protocols and data dictionaries for the sampled datasets were available to users without the need to submit a formal access request.

## RESULTS

### Platform selection

A total of seven U.S. clinical trial data-sharing platforms were examined: CSDR, Vivli, Biolincc, dbGaP, PDS, Data Share, and DASH. CSDR is a general clinical trials data-sharing platform funded by consortium fees that had 3,722 dataset records as of April 2019. Vivli is a general clinical trials data-sharing platform funded by membership fees and grants that had 3,885 records as of May 2019. Biolincc is a general clinical trials data-sharing platform funded by taxpayers that had 204 records as of May 2019. dbGaP is a genetics clinical trials data-sharing platform funded by taxpayers that had 1,140 records as of May 2019. PDS is an oncology clinical trials data-sharing platform funded by taxpayers that had 191 records as of May 2019. Data Share is a drug abuse clinical trials data-sharing platform funded by taxpayers that had 73 records as of May 2019. DASH is a child health and human development clinical trials data-sharing platform funded by taxpayers that had 7,931 records as of May 2019.

### Dataset previews and access

“Level of review” refers to the extent to which users can preview trial datasets before needing to formally request access. “Preview” indicates that users may only access a summary description and/or preview of the dataset before needing to make a formal access request, whereas “immediate download” indicates that the full dataset can be downloaded without a formal access request. All platforms provided at least some level of review for their datasets. Data Share had the highest level of review, with 100% of sampled datasets granting users complete access without the need for a formal access request (Table 1).

**Table 1:** Platform Dataset Previews

platform	level of review	data format preview	request level	downloadable
CSDR	preview	no	batch	no
Vivli	preview	no	batch	no
Biolincc	preview	no	single	yes
dbGaP	preview	no	single	yes
PDS	preview	yes	batch	sometimes
Data Share	immediate download	yes	single	yes
DASH	preview	yes	single	yes

“Data format preview” indicates whether data format information is provided in the dataset preview (e.g., whether the dataset is downloadable as a CSV file, SAS file, etc.). PDS, Data Share, and DASH were the only platforms that provided data format information in their previews.

“Request level” refers to whether a single request can be submitted to access multiple datasets (“batch”) or whether each dataset requires its own access request (“single”). CSDR, Vivli, and PDS allowed for “batch” request of datasets, whereas the other platforms required requests at the “single” dataset level.

“Downloadable” indicates whether datasets can be downloaded onto a personal device (“yes”) or only accessed in the platform's environment (“no”). Biolincc, dbGaP, Data Share, and DASH allowed the download of all sampled datasets, and PDS allowed the download of some sampled datasets (while other PDS datasets were only accessible via PDS's secure online environment). By contrast, CSDR and Vivli only allowed access to datasets within their platform's secure online environments (Table 1).

“Access requirements” refer to the extent to which platforms restrict access to datasets. All platforms required users to sign a data use agreement to access datasets. Data Share had the fewest request walls, with the only requirement being that users had to click to sign a data use agreement prior to accessing a dataset. Vivli was the only platform that charged a fee to access datasets, related to its requirement for accessing data within the platform's secure online environment (Table 2).

**Table 2:** Platform Access Requirements^*^

platform	request walls	pay to access?
CSDR	A, P, D	no
Vivli	A, P, D	indirect fee
Biolincc	A, P, D	no
dbGaP	A, P, D	no
PDS	A, P, D	no
Data Share	D	no
DASH	A, P, D	no

* A = account required; P = project proposal required, D = data use agreement required

### Links to ClinicalTrials.gov and other resources

“Link to ClinicalTrials.gov” indicates the number of each platform's sampled dataset previews with a link to their associated trial record in ClinicalTrials.gov. “Link from ClinicalTrials.gov” indicates the number of dataset previews that had a link from their associated trial record in ClinicalTrials.gov. Except for dbGaP, most or all dataset previews were linked to trials in ClinicalTrials.gov. However, only Biolincc had some dataset previews with a link from their associated trial record in ClinicalTrials.gov (Table 3).

**Table 3:** Platforms' Links to ClinicalTrials.gov*

Platform	Link to ClinicalTrials.gov	Link from ClinicalTrials.gov	Result posted	Registered
CSDR	10	0	5	10
Vivli	10	0	4	10
Biolincc	9	5	5	9
dbGaP	0	0	0	0
PDS	10	0	8	10
Data Share	10	0	0	10
DASH	9	0	1	9

* CND = could not be determined

“Results posted” refers to whether the results of the trials associated with the sampled datasets were posted on ClinicalTrials.gov, and “Registered” refers to whether the trials associated with the datasets were registered on ClinicalTrials.gov. PDS had the largest number of sampled datasets from trials that posted results and were registered on ClinicalTrials.gov. All platforms except dbGaP had most of their sampled trials post registry information on ClinicalTrials.gov (Table 3).

CSDR, Vivli, Biolincc, dbGaP, and DASH had links to resources apart from ClinicalTrials.gov. The most common such links were to study websites and associated publications (Table 4).

**Table 4:** Platforms' Links to Other Resources

platform	sponsor website	study website	publication	other
CSDR	0	0	0	10
Vivli	1	0	0	0
Biolincc	0	3	6	0
dbGaP	0	1	6	8
PDS	0	0	0	0
Data Share	0	0	0	0
DASH	0	5	8	0

### Availability of protocols and data dictionaries

“Protocol available” indicates the number of sampled dataset previews that had evidence of an available protocol, and “protocol downloadable” indicates whether these protocols could be downloaded without needing to make a formal access request. Most sampled dataset previews in CSDR, Biolincc, Data Share, and DASH had evidence of protocols available (Table 5); however, only Biolincc, Data Share, and DASH provided complete access to these protocols without the need for a formal access request.

**Table 5:** Platform Protocol Availability*

platform	Protocol Available	Protocol Downloadable
CSDR	9	no
Vivli	CND	no
Biolincc	10	yes
dbGaP	CND	no
PDS	CND	no
Data Share	10	yes
DASH	9	yes

* CND = could not be determined

“Data dictionary available” indicates the number of sampled dataset previews that had evidence of an available data dictionary, and “data dictionary downloadable” indicates whether these data dictionaries could be downloaded without making a formal access request. Dataset previews on all platforms except CSDR and Vivli indicated that a data dictionary was available (Table 6). Also, most dataset previews in all platforms except CSDR and Vivli allowed users to download data dictionaries without a formal access request. The most common data dictionary file types were PDF, XML, and Excel formats.

**Table 6:** Data Dictionary Sharing

platform	Data dictionary available	xml	zip	pdf	excel	word	Undetermined	Data dictionary downloadable
CSDR	9	0	0	0	0	0	9	0
Vivli	4	0	0	0	0	0	4	0
Biolincc	10	0	0	10	0	0	0	10
dbGaP	10	10	0	0	0	0	0	10
PDS	10	0	0	7	2	1	0	8
NIDA	10	0	0	0	10	0	0	10
DASH	10	0	6	9	7	0	0	10

## DISCUSSION

While many studies examined barriers to sharing data within clinical trials data-sharing platforms and, to an extent, barriers to data re-use, few performed specific, in-depth evaluation of preview information within clinical trials data-sharing platforms. Banzi et al.'s 2019 study, which evaluated the suitability of 25 clinical data-sharing platforms for hosting clinical trials data, included metadata availability as one of many criteria and found that only 12 platforms demonstrated the presence of sufficient metadata [15]. In this study, we examined the availability of metadata (referred to as “preview information”) with greater granularity and through the perspective of a data reuser rather than a data sharer. We hope our findings spark discussions about, and future studies into, the degree to which preview information is available within clinical trials data-sharing platforms, which has the potential to enhance the discovery and utility of clinical trials data for data reusers. Similar to Banzi et al.'s 2019 observations of metadata availability, we found that preview information varied between platforms and sometimes between datasets within platforms.

We found that the extent to which users can preview clinical trial datasets across the seven selected platforms was fairly good. All platforms provided some degree of dataset preview, but only Data Share allowed the immediate and unrestricted download of datasets. The prevalence of data previews, as opposed to complete access to datasets, reflects the “openness versus security” balance that clinical trial data-sharing platforms must strike, wherein the openness of data must be balanced with providing enough protections to safeguard sensitive information [10]. These results may also reflect findings from surveys of researchers' reservations about, or barriers to, data sharing. A Figshare 2019 State of Open Data survey of 8,000-plus participants from more than 190 countries revealed “that over 2,000 respondents had concerns about misuse of their research data” [22]. Such reservations included, among other things, fears of data reusers misinterpreting shared data, compromising participant privacy, and conducting misleading or inappropriate secondary analyses [22]. Other studies of researcher reservations about data sharing report similar findings [6, 23]. Having an access wall accompanied by a preview, rather than allowing for the unrestricted download of data, is one way of maintaining some level of control over how datasets may be reused [24].

Of the platforms, only PDS, Data Share, and DASH had data format information in their sampled dataset previews (e.g., whether datasets were available as CSV files, SAS files, etc.). The absence of this information in dataset previews could be attributed to multiple factors, with the most simple explanation being that standardized formatting requirements could be provided in an alternative location on the platform. However, especially with the increase in studies that combine multiple datasets, it would be beneficial to provide this information in dataset previews to quickly inform users about the interoperability of the datasets and their suitability for subsequent research.

Modes of data transfer, such as requesting multiple datasets and the environment in which datasets may be accessed, were largely split among platforms. Three of the seven platforms appeared to allow batch downloading of datasets, whereas four platforms required researchers to submit a separate request for each dataset. Also, four of the seven platforms allowed users to download all available datasets onto personal devices, whereas three platforms only allowed users to access datasets in platform-specific online environments. Requiring researchers to submit separate data requests and limiting data access to platform-specific environments may reflect platforms' and researchers' fears of violating patient privacy, as these measures would allow for greater control and security of data; however, these additional hurdles may discourage researchers from reusing a dataset [25]. While information on whether users may download datasets onto personal devices was readily available on each of the platforms' websites, information on whether datasets could be requested on a batch level was more difficult to find. In most cases, we could only verify this information by directly attempting to download the datasets in batches or requesting multiple datasets in a single batch request. With an increasing number of projects dependent on combining multiple datasets, presenting this information on the platform home page could save users valuable time in determining how to most effectively use a given platform.

In terms of data access, Data Share presented the fewest request walls before users could access a dataset, requiring users only to acknowledge the stipulations outlined in an agreement. This is a particularly convenient feature, as users would not need to wait for review panels to approve their request and could acquire instant access to datasets. The use of request walls by other platforms, however, is not unexpected, as repositories must continuously balance the need to provide open access to datasets while protecting sensitive information from trial participants [10]. Additionally, the mode of data access depends greatly on the exact language used in the informed consent form signed by participants during trial enrollment and whether a platform devotes resources to perform data de-identification. However, as noted by Shebani et al., such controlled access models, especially if the models are administratively heavy and excessive in their requirements, can increase the burden placed on users and may dissuade them from requesting access to datasets [24].

We found that datasets in clinical trial data-sharing platforms often linked out to other resources, allowing users to discover additional background information associated with the trials. However, links from ClinicalTrials.gov to the platforms could be considerably improved. Although all platforms except dbGaP consistently linked out to trial records in ClinicalTrials.gov, Biolincc was the only platform to which ClinicalTrials.gov consistently linked back. This absence of links from ClinicalTrials.gov to dataset records in clinical trial data-sharing platforms was rather surprising, as ClinicalTrials.gov is the largest clinical trial registry, containing records for 306,775 trials from 210 countries as of May 2019 [16]. The fact that most ClinicalTrials.gov records for sampled trials provided no mention of where their data could be accessed may severely impede the discoverability of those data, especially for users who rely on ClinicalTrials.gov as a primary resource for finding clinical trials of interest. More research should be performed to gain insight into the extent to which ClinicalTrials.gov records exclude linkages to locations where datasets may be accessed.

Another concern is the inconsistent posting of trial results on ClinicalTrials.gov. Only datasets in PDS were associated with ClinicalTrials.gov records that had trial results posted with some consistency; only half of datasets in other platforms were associated with ClinicalTrials.gov records with trial results posted. In other words, trial datasets were available on a clinical trial data-sharing platform, but their ClinicalTrials.gov record indicated no trial results. This aligns with findings from a 2015 study that found a low percentage of results reporting even among studies that were likely subject to Food and Drug Administration Amendments Act requirements for timely reporting of clinical trial results to ClinicalTrials.gov (13.4% of trials reported summary results within 12 months after trial completion, and 38.3% at any time after trial completion) [18]. This lack of results reporting on ClinicalTrials.gov could impede the discoverability of clinical trials data. If users were to locate a trial on ClinicalTrials.gov that did not post its results, they may assume that the trial was incomplete or terminated before completion. Providing a link from ClinicalTrials.gov records to dataset records on clinical trial data-sharing platforms and posting summative results on ClinicalTrials.gov in a timely manner could resolve these issues.

Clinical trial data-sharing platforms could also better standardize the extent to which their records link out to other resources. Only dbGaP, DASH, and Biolincc provided links to associated study websites and primary publications. Consistently linking out to these materials could greatly improve the reuse value of a dataset and consequent citation of its associated publications, while also facilitating the contextualization of the dataset and its associated materials [17, 26].

Four out of seven platforms provided evidence of the availability of trial protocols; however, only three platforms provided protocols that were directly downloadable (i.e., they did not require a formal request to access the protocol). These findings reflect a 2017 study that found that the availability and discoverability of clinical trial protocols were generally suboptimal [21]. The inaccessibility or potential inexistence of these protocols severely restricts users' ability to appraise a dataset prior to making a formal data request and raises the question of whether protocols contain enough sensitive information to warrant these request walls.

Six of the seven platforms provided evidence of the availability of data dictionaries. Five platforms also provided information on the file formats of the data dictionaries for most sampled datasets. These findings are promising, as they indicate that platform designers are attributing high importance to the inclusion of data dictionaries and their file formats. The interoperability of data dictionary file formats, however, was less promising. dbGaP and Data Share had data dictionaries that could consistently be downloaded in a machine-readable format (e.g., XML or Excel); in contrast, formats such as PDF often require human processing before they can be used by computer algorithms. File formats that are not machine readable (e.g., PDFs) can be a great burden to researchers, as they can require significant processing to make the associated data machine readable [27]. CSDR and Vivli were the only platforms that did not allow downloading of any data dictionaries without a formal access request. This observation is interesting, as data dictionaries do not contain individual participant data and, like protocols, are unlikely to contain sensitive information.

This study provides a limited but insightful view of the current landscape of information provision on clinical trial data-sharing platforms. Due to the exploratory nature of the study and time constraints, we evaluated only 10 non-representative dataset records on each of 7 selected platforms, although we attempted to include the most recently submitted datasets. As we often found variability in the extent of information provision within platforms, it is likely that some characteristics of a platform's information provision may not be well-represented in our convenience sample. However, this sample may be reflective of how unfamiliar users may quickly evaluate clinical trial data-sharing platforms. Also, our results are limited to the information we could find on the platforms, and it is possible that some information was missed or misinterpreted. Again, however, this may reflect the difficulties experienced by dataset submitters or users when attempting to locate similar information on the platforms.

In conclusion, increasing the amount of information provided in dataset previews, including access to dataset details, protocols, data dictionaries, and cross-links to ClinicalTrials.gov and other external resources, could considerably improve the discoverability and utility of datasets on clinical trial data-sharing platforms. Our results suggest that access to this information could be improved and would serve to enhance the discoverability of clinical trial data.

## Data Availability

The data, data dictionary, and README files associated with this article are available in GitHub at https://github.com/weepai/Clinical-Trial-Data-Sharing-and-Platforms-Evaluation-Data
